# Cares and Coffins

**Published:** 1890-04-12

**Authors:** 


### The Doctor's Pulpit.

### CARES AND COFFINS.

"Care to our coffin adds a nail, no doubt;
And every grin, so merry, draws one out."

The idea of a man alternately making and unmaking his own coffin does not belong to the cheerful order of mental pictures; though there is no doubt that when John Wolcot penned his expostulary lines he was fully minded "to drive dull care away." The winter to which we are now bidding farewell—a farewell that some of us would willingly transform into a parting kick—has been by no means a merry season. The Influenza fiend, whose terrors we all made so light of last November, has proved his power to fill the air with such volumes of pestilential sulphur-smoke as have been neither comfortable nor safe. Hardly a single household, in London at any rate, has altogether escaped the stroke of his rushing wings. Many contemptuous sceptics have been laid low, and a few, who probably had not incurred the guilt of unbelief, have nevertheless paid the last debt of nature at the stranger's bidding. The season of festivities, of eating and drinking and dancing and merry-making, has been dull; it still is dull indeed. March usually brings us sunshine and clear skies, even if they be accompanied by cold and biting winds. This year we have not had the sunshine. The winds may not have been quite so biting as usual, but they have been mean little airs that have not met you full in the face with a loud and boisterous roar, but have sneaked round corners and caught you by the throat, or dropped down from roofs, insinuating themselves between the neck and the coat collar with moist malignity. It cannot, with strict truth, be denied that March "came in like a lion," or even that it "went out like a lamb." But then it went out like a dull, spiritless lamb that has been prematurely washed in some vile "sheep-dipping composition," a lamb to which frolic and fun were the very last things to be thought of.

But the miserable month has gone, and we will all be thankful for that, even though we are a whole year older than we were in March, 1889. The skies are looking a little less like a universal pall of lead. We can convince ourselves by ocular demonstration, at some period or other of the twenty-four hours of the day, that the sun is still in existence. The birds, it is probable, have been visited by the Influenza epidemic, for they look limp and debilitated, and hardly sing at all. Perhaps the Fellows of the Feathered College of Physicians have been like their bimanal confrères, and have not understood epidemic Influenza and its treatment very well. Be that as it may, the spring buds are mostly at the bursting point, whilst the birds seem to be still muffled up in their thickest winter clothing.

But no season of depression can last for ever. The deadliest of typhoid fevers must either kill its victim or land him in convalescence sometimes. The longest winter, the dullest weather, the rainiest clouds, the most persistent bronchitis, each and all know some kind of limit. Spring must follow winter, and summer succeed spring, as inevitably as morning treads on the heels of midnight. No scientist, however profound, has ever been able to see any visible effort made by nature at the spring period of new and bursting life. Yet the results of the spring-time are so marvellous in themselves, and on such a world-wide scale, that the human creature, accustomed as he is to making efforts almost painful in their strenuousness when he has great work on hand, cannot think it possible that even nature herself can burst the bonds of winter and rush forth with all the various and intense energies of spring without an adequate degree of strenuous, though apparently unconscious, effort. Nature is new as well as old in her work, original as well as conservative. She is appealed to here in order that her authority and example may be claimed for an appeal we are about to make to men and women.

Have human beings any considerable power of lightening their own loads in life? Can an ordinary man make his life any happier by his own personal efforts? Is there a spring-time and a summer of the body and the mind and the soul as well as of the trees and the cornfields? The heedless man essays to "drive dull care away" by means of his glass, and his pipe, and his boon companions; and for a brief hour or two he is no doubt perfectly successful. Are there any means whereby the unnatural light-heartedness of the merry evening can be made natural and carried throughout the day and the lifetime? These, it will be observed, are fundamental questions; they are not new. They will not, perhaps, attract any very great attention at the hands of readers, because most readers probably think there is only one answer to them, and that of a hopeless and very dreary character.

We frankly confess our belief in effort. Men, as men, are not under the necessity of being beaten, enslaved, and miserable. Notwithstanding Carlyle's protest against man's hunger for happiness, we cannot but express the conviction that happiness is possible—that it is indeed one of the leading purposes of nature to make men happy. Why then are they not happy? Why do men feel themselves so powerless, and hopeless in the midst of the millions of their fellow-men and of the complex problems of civilization? Many of us think that if we only had a competency, and were independent of our fellows, we should be happy. Perhaps we should. But the problem that is before us is not how can a man be happy with a competency? but how can he be happy without a competency; without indeed any stored resources at all? Is this problem capable of a cheerful solution? Certainly! The great demand that nature makes upon all men, in all periods, is that they shall understand their own epoch and their own surroundings, and that they shall adapt themselves intelligently and victoriously thereto. Stupidity is misery; cowardice is misery; a wilful quarrel with one's surroundings and circumstances is misery. Understanding, courage, sympathy with nature in her highest and lowest moods, a resolution that nothing can bend or break—these are the materials out of which a palace of happiness may be built.

It is possible to strangle care, and to carry out his dead body to the burial. But how? Effort, permanent effort, is the prime condition. Permanent effort; because care, even when dead and buried, gives origin to a numerous progeny of ghosts. But mere blind effort will effect nothing; it may even make the miserable striver fall into deeper depths and abysses more profound. These three faculties, at least, must make a simultaneous effort: the judgment, the courage, and the will. The judgment, as well as the courage and the will: for no physician will cure a disease unless he goes to the root of the matter, and finds out the cause. So must the miserable man search for the cause of his misery, and look it full in the face. Is it straitened means? Then he must see what can be done to increase his means, or to diminish his wants. Perhaps it is physical health that is at the root of another man's misery! Can this be overcome? In many cases it undoubtedly can. But the condition of its vanquishment is the difficulty. Thousands of people invoke the doctor's aid for health when they ought to invoke their own. The art of healthy and happy living is like the art of navigation. It consists in adapting your ship to the water she has to sail in and to the freight she has to carry, and in taking advantage of every favourable circumstance of wind and tide that makes for a prosperous voyage. The spring and the summer are such favourable circumstances in the voyage of the health navigator. But their value must be understood and taken due advantage of. Nobody can get all the vitality that is stored up in spring and summer who confines himself to the atmosphere and habits of town life. We must put ourselves in the way not only of securing the pure breezes of hill-top and sea, but also of quickening our bodily movements, varying our pleasures, diminishing the extent and strenuousness of our labours. Men have to make up their minds that holidays are good things, and that they are to be real pleasures. They have, in short, to remember that life in these times, like business, is exceedingly complex; and that also, like business, life and health must be carefully "financed" or bankruptcy will be the result.

